# Nonconstrictive epicarditis mimicking a cardiac mass in a 71-year-old Caucasian man: a case report and review of the literature

**DOI:** 10.1186/1752-1947-3-2

**Published:** 2009-01-06

**Authors:** Asa M Margolis, Andrew B Emmerman, Mario Rascon, Saima I Chaudhry

**Affiliations:** 1 Department of Medicine North Shore University Hospital, 300 Community Drive, Manhasset, New York 11030, NY, USA; 2 Department of Pathology, North Shore University Hospital, 300 Community Drive, Manhasset, New York 11030, NY, USA

## Abstract

**Introduction:**

Isolated cases of epicarditis are rare. Thus far, all have occurred with constrictive physiology as most cases involve both parietal and visceral pericardium. We report the first case of asymptomatic epicarditis that involved only the visceral pericardium presenting without constrictive physiology.

**Case presentation:**

A 71-year-old male with a history of atrial fibrillation, coronary artery disease, pericardial effusion, type-2 diabetes and hypothyroidism presented with 5 weeks of fatigue and 1 day of dizziness. Physical examination was significant for pallor and tachycardia. Laboratory analysis revealed a hemoglobin count of 7.2 g/dl and iron deficiency anemia. The patient was transfused and evaluated by endoscopic ultrasound. A polypoid mass in the gastric cardia was found and later diagnosed as gastric adenocarcinoma (staged as T1N0M0). The pericardial effusion was evaluated with transthoracic echocardiography which showed a 2.0 × 2.7 cm mass associated with the right atrium. Transesophageal echocardiography confirmed the mass but did not reveal constrictive physiology. Whole-body contrast computed tomography failed to demonstrate metastatic disease. Biopsy of the cardiac mass revealed epicarditis without parietal pericardium involvement. Partial gastrectomy was performed to remove the gastric adenocarcinoma.

**Conclusion:**

This is the first reported case of asymptomatic epicarditis. Our case was especially unusual because the epicarditis presented as an incidental cardiac mass. The clinical picture was complicated due to the concomitant presence of gastric adenocarcinoma and chronic pericardial effusion. This case demonstrates that epicarditis should be considered in the differential diagnosis of cardiac masses.

## Introduction

Epicarditis, inflammation of the visceral epicardium, occurs very rarely. Most often, cases of epicarditis occur concurrently with both parietal pericardium involvement and constrictive physiology. In these reports, epicarditis was most often diagnosed after pericardiectomy failed to alleviate the patient's symptoms. However, isolated cases of exclusive epicarditis without involvement of the parietal pericardium or myocardium have been reported.

We report the first case of asymptomatic effusive epicarditis without involvement of the parietal pericardium. In our patient, epicarditis presented as a cardiac mass occurring synchronously with newly diagnosed gastric adenocarcinoma. To our knowledge, prior cases of epicarditis have not occurred in the setting of a malignancy and only one prior patient presented with a cardiac mass. The uniqueness of our case is illustrated by contrasting our patient with prior reports of epicarditis with the emphasis on patient presentation, presence of constrictive physiology, method of diagnosis and suspected etiology. We do not include cases of epicarditis that occurred as a consequence of traumatic injury, thoracic surgery or neonatal cases.

## Case presentation

A 71-year-old Caucasian man with a past medical history significant for type-2 diabetes, coronary artery disease, atrial fibrillation, chronic pericardial effusion and hypothyroidism presented with 5 weeks of increasing fatigue and 1 day of dizziness.

Physical examination revealed that the patient was afebrile, had a blood pressure of 140/90 mmHg, an irregularly irregular heart rate of 102 beats per minute and a respiratory rate of 16 breaths per minute. There was no pulsus parodoxus. Examination of the head and neck showed pale conjunctiva, no palpable lymphadenopathy, jugular venous distension or bruits. Auscultation of the chest revealed scattered rhonchi. The patient was mildly tachycardic with no S3 or S4 heart sounds, murmurs or rubs appreciated. There was no hepatosplenomegaly, clubbing, cyanosis or edema and stools were guaiac negative.

An electrocardiogram (EKG) on admission demonstrated atrial fibrillation with a rapid ventricular response with low voltage QRS complexes; electrical alternans were not present. The chest radiograph showed a very minimal pleural effusion with no cardiomegaly, infiltrates or masses.

Laboratory analysis revealed a white blood-cell count of 12,900/L, with a normal differential, and a hemoglobin and hematocrit of 7.2 g/dl and 23.4%, respectively. The platelet count was 387 thousand/L. The red cell indices were microcytic and iron studies revealed an iron deficiency anemia. Liver function tests were normal. The lactate dehydrogenase (LDH) was 167 U/L (within normal limits). The thyroid-stimulating hormone (TSH) was 16.49 mcIU/ml and the free T3 was 57 mcg/ml.

The patient was admitted for anemia and worked up for a gastrointestinal bleed. Three units packed red blood cells were transfused. Upper endoscopy revealed a mass just below the gastroesophageal junction and three nonbleeding gastric ulcers. To further evaluate the gastric mass, an endoscopic ultrasound (EUS) was performed demonstrating a 30 × 17 mm polypoid mass visualized in the gastric cardia limited to the mucosa/submucosa. The biopsy revealed gastric adenocarcinoma, staged as T1N0M0 by EUS, and was *Helicobacter pylori*-negative.

**Figure 1:**
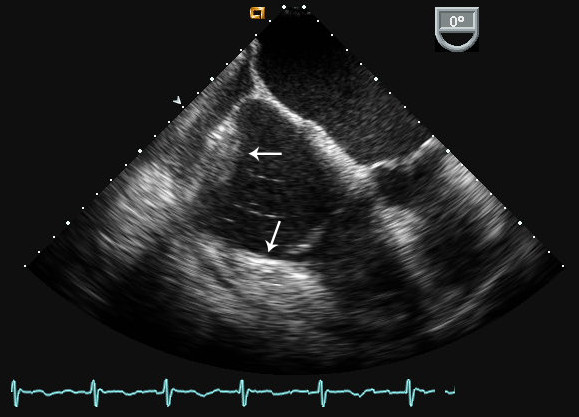
**Transesophageal echocardiogram (midesophageal view) with echodensities in the right atrial free wall, right ventricular free wall and atrioventricular groove (arrows)**.

**Figure 2:**
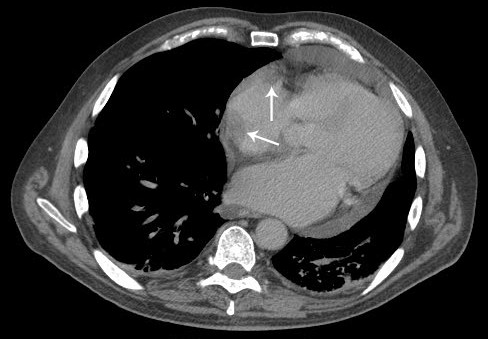
**Contrast-enhanced computed tomographic axial image demonstrating filling defects (arrows) corresponding to echocardiographic findings**.

A two-dimensional echocardiogram was preformed to evaluate an 8-month old pericardial effusion. It demonstrated a 2.0 × 2.7 cm mass associated with the right atrium (RA). To better define the mass and its anatomic relationship within the RA, several imaging procedures were performed: 1) transesophageal echocardiogram (TEE) showing a 5.7 cm^2 ^shaped echodensity within the wall of the RA and a second, 16 cm^2 ^echodensity, that extended across the atrioventricular groove (Figure 1); 2) a contrast computed tomography (CT) of the chest, abdomen and pelvis negative for metastasis, but significant for a large pericardial effusion without pericardial thickening and a 2.6 × 2.1 cm filling defect in the right atrial appendage (Figure 2); and 3) a cardiac MRI demonstrating a homogenous enhancing mass along the lateral wall of the right atrium. A positron emission tomography (PET) scan demonstrated a hypermetabolic region in the heart corresponding to the mass observed on CT.

At this point, our differential diagnosis included a primary gastric adenocarcinoma with metastasis to the heart as well as two separate primary neoplastic processes (one involving the heart and one involving the stomach).

**Figure 3:**
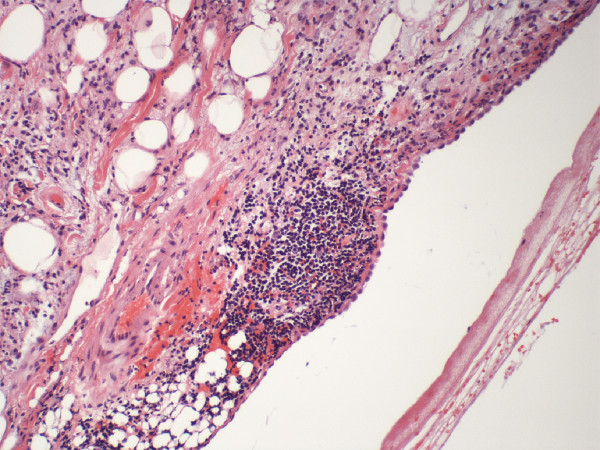
**High power histologic examination showing the epicardium with lymphoplasmocytic infiltration (hematoxylin-eosin stain)**.

To determine the etiology of the cardiac mass, a biopsy was accomplished via a pericardial window through an anterolateral thoracotomy. At thoracotomy, the parietal pericardium appeared normal. Visual inspection revealed several nodular areas over the body of the right atrium, superior vena cava (SVC) and inferior vena cava (IVC). There was no evidence of a thickened, constrictive layer surrounding the heart. Two hundred milliliters of straw-colored fluid was recovered from the pericardial cavity. The fluid was negative for malignant cells and consisted of a few benign and reactive mesothelial cells mixed with inflammatory cells and proteinaceous debris. Viral, bacterial and mycobacterial cultures of the pericardial fluid were negative. Biopsy revealed a normal parietal pericardium and myocardium. However, the visceral pericardium showed a lymphoplasmocytic infiltrate diagnostic of epicarditis (Figure 3). After thoracotomy, the patient had a partial gastrectomy to remove the adenocarcinoma. The postoperative course was complicated by infection, requiring a 3-month stay in the intensive care unit. During this time, there was no evidence suggestive of constrictive physiology. Since the patient continued to remain asymptomatic from the epicarditis, no further imaging studies or procedures were performed to follow the "mass." The patient eventually succumbed to infection.

## Discussion

Our case presents several significant and rare findings: 1) epicarditis without involvement of the parietal pericardium; 2) epicarditis occurring without constrictive physiology; 3) epicarditis mimicking a cardiac tumor; and 4) epicarditis occurring with gastric adenocarcinoma.

The co-existence of a pericardial effusion and constrictive epicarditis was first described by Burchell [1] and Edwards [2] in 1954. Since then, there have been reported cases of epicarditis associated with various medical conditions (Tables 1 and 2) [3-11]. The natural history has been described as sequential, with progression from subacute effusive constrictive epicarditis to chronic constriction without effusion and, ultimately, the possibility of myocardial infiltration [4]. To our knowledge, this is the first report of asymptomatic epicarditis without parietal pericardial involvement. Only two cases of exclusive epicarditis (one presenting as a mass) have been reported previously and both occurred in the setting of symptoms and constrictive physiology. These patients had a grossly and microscopically normal parietal pericardium (Table 1). There is the possibility that other cases of asymptomatic epicarditis have occurred in patients. However, these individuals would not have presented in a manner to warrant investigation for such a diagnosis. Thus, we looked into whether any post-mortem studies have addressed this as a finding. There was no evidence of clinicopathological studies or post-mortem cardiac examination reporting a dense fibrous epicardium or diagnosis of epicarditis.

**Table 1:** Cases of Exclusive Epicarditis without Parietal Pericardial Involvement*

Case [Reference]	Age (y) Sex	Symptoms	Physical Exam Findings	Constrictive Physiology	Gross ± Microscopic Pathology of Epicardium	Parietal Pericardium Involvement	Echocardiogram/CT	Suspected Etiology
**1** [3]	22 M	Dyspnea, anorexia, weight loss	S3 heart sound, hepatomegaly, anasarca	+	Hyaline thickening with sparse mononuclear infiltration	0	TTE: Large pericardial effusion	-
**2** [4]	16 M	Dyspnea, abdominal distention	Ascites, hepato-splenomegaly, pedal edema	+	Taught white membrane 2 mm thick	0	-	Probably viral
**3** (Our case)	71 M	Fatigue, weight loss	None	0	Lympho – plasmocitic infiltrate	0	TEE: Two echodense masses, circumferential pericardial effusion/ CT: Filling defect in right atrial appendage	-

*Table does not include cases of epicarditis as a consequence of traumatic injury, thoracic surgery, or neonatal cases.

CT, Computed tomography; TEE, Transesophageal echocardiogram; TTE, Transthoracic echocardiogram

**Table 2:** Cases of Epicarditis with Parietal Pericardial Involvement*

Case [Reference]	Age(y) Sex	Symptoms	Physical Exam Findings	Constrictive Physiology	Gross ± Microscopic Pathology of Epicardium	Parietal Pericardium Involvement	Echocardiography/CT	Suspected Etiology
1 [5]	83 F	Dyspnea on exertion	JVD, generalized edema, hepatomegaly	+	Dense, calcified, ossified epicardial thickening	+	CT: Calcification ring encircling the ventricle	-
2 [4]	25 M	Fever, dyspnea, chest pain	Muffled heart sounds, hepatomegaly	+	Taught white membrane 7 mm thick	+	-	Tuberculosis
3 [4]	45 M	Fever, dyspnea	Muffled heart sounds, Kussmaul's sign, hepatomegaly	+	Taught white membrane 8 mm thick infiltrating into myocardium	+	-	Tuberculosis
4 [4]	17 F	Fever, orthopnea, chest pain	Muffled heart sounds, hepatomegaly, pedal edema	+	Taught white membrane 10 mm thick	+	-	Acute pyogenic infection
5 [4]	21 F	Fever, orthopnea, chest pain	Pericardial rub	+	Taught white membrane 3 mm thick	+	-	Probably viral
6 [6]	33 M	Pleuritic chest pain, fever, fatigue	Hepatomegaly, pitting ankle edema	+	Myocardium bulging through hole in epicardium	+	TTE: Anterior and posterior pericardial effusion	Coxsackie virus
7 [7]	10 mo M	-	JVD, muffled heart sounds, hepatomegaly	+	Thickened epicardium	+	-	Acute Staphylococcus osteomyelitis of left humerus
8 [8]	51 M	Dyspnea, fatigue	JVD, Kussmaul's sign, pedal edema	+	Thickened with marked fibrosis and hyalinization	+	TEE: Thickened visceral pericardium	Associated with ASD
9 [9]	13 M	-	JVD, ascites, peripheral edema	+	Diffusely thickened	+	-	Staphylococcal sepsis
10 [9]	41 M	-	JVD, ascites, peripheral edema	+	Diffusely thickened	+	-	-
11 [9]	36 M	-	JVD, ascites, peripheral edema	+	Diffusely thickened	+	-	Tuberculosis
12 [9]	73 F	-	JVD, ascites, peripheral edema	+	Constrictive sclerosis	+	-	-
13 [10]	24 M	Dyspnea	Hepatomegaly, JVD, peripheral edema	+	Thickened and constricting, noted to be densely adherent to myocardium	+	-	Infectious mononucleosis
14 [11]	53 M	Fatigue	Ascites and pedal edema	+	Taught, 3 – 5 mm thick	+	-	-

*Table does not include cases of epicarditis as a consequence of traumatic injury, thoracic surgery, or neonatal cases.

CT, Computed tomography; JVD, Jugular venous distention; TEE, Transesophageal echocardiogram; TTE, Transthoracic echocardiogram

Our case highlights the importance of cardiac biopsy in the differential diagnosis of cardiac masses. Despite the absence of metastasis on radiographic imaging, the possibility that the cardiac mass represented a metastatic adenocarcinoma, or a second malignant process, necessitated the need for biopsy. Cardiac biopsy with immunohistochemical staining was instrumental in determining the diagnosis of epicarditis.

Prior case reports have suggested the etiology of epicarditis to be infectious in origin, including reports citing viral, bacterial and mycobacterial causes. However, in many of the case reports, no cause was reported. Although there have been no reports of epicarditis occurring as a paraneoplastic phenomenon associated with any type of malignancy, there are few reports of gastric carcinoma occurring with paraneoplastic syndromes. We raise the possibility that our patient's gastric adenocarcinoma created an inflammatory milieu resulting in a localized, focal inflammatory response in the epicardium mimicking a cardiac mass. The biological basis for this paraneoplastic phenomenon may be similar to other paraneoplastic processes in which there is the elaboration of interleukin-6 (IL-6) as well as growth factors produced by the tumor [12]. While gastric cancer is not a malignancy often associated with paraneoplastic syndromes, there are documented dermatologic findings suggested as systemic manifestations related to a paraneoplastic phenomenon, including dermatomyositis [13]. Further, there are other paraneoplastic conditions that have been demonstrated to occur with gastric cancer including reports of Anti-Yo-associated paraneoplastic cerebellar degeneration [14] and palmar fasciitis and polyarthritis [15].

## Conclusion

Our case highlights several important points: 1) epicarditis can be asymptomatic and can occur without involvement of the parietal pericardium; 2) it can mimic a cardiac mass; 3) cardiac biopsy is essential for diagnosis; and 4) although most reports have suggested infection as the etiology, we raise the possibility of a paraneoplastic syndrome creating the epicarditis. To that end, further studies should investigate this hypothesis.

Once the diagnosis is established, epicarditis should be treated on a case-by-case basis based on patient symptomatology and expectant morbidity. If constrictive physiology develops, one must distinguish whether it is the result of parietal and/or visceral pericardial involvement. Treatment should then be directed at removal of the involved layer.

## Abbreviations

EKG: electrocardiogram; LDH: lactate dehydrogenase; TSH: thyroid-stimulating hormone; TEE: transesophageal echocardiogram; CT: computed tomography; EUS: endoscopic ultrasound; RA: right atrium; PET: positron emission tomography; MRI: magnetic resonance imaging; SVC: superior vena cava; IVC: inferior vena cava

## Consent

Written informed consent was obtained from the next of kin of the patient for publication of this case report and accompanying images. A copy of the written consent is available for review by the Editor-in-Chief of this journal.

## Competing interests

The authors declare that they have no competing interests.

## Authors' contributions

AM, AE, and SC were all involved in the conception of the case report, data collection, review of literature and writing the manuscript. MR participated in data collection and in rendering a pathological diagnosis. All authors read and approved the final manuscript.
